# Supplementary material for: Identification of Regions Involved in the Physical Interaction between Melanocortin Receptor Accessory Protein 2 and Prokineticin Receptor 2
Source: Biomolecules. 2022 Mar 20;12(3):474. doi: 10.3390/biom12030474 (PMC8946000; doi:10.3390/biom12030474)

Original Images of Western Blots

Identification of regions involved in physical interaction between Melanocortin Receptor Accessory Protein 2 and Prokineticin Receptor 2

Maria Rosaria Fullone[1], Daniela Maftei[2], Martina Vincenzi[2], Roberta Lattanzi[2], Rossella Miele[1]*

[1]Department of Biochemical Sciences "Alessandro Rossi Fanelli", Sapienza University of Rome, Piazzale Aldo Moro 5, I-00185 Rome, Italy
[2]Department of Physiology and Pharmacology "Vittorio Erspamer", Sapienza University of Rome, Piazzale Aldo Moro 5, I-00185 Rome, Italy

Figure S1. Original western blots of figure 1

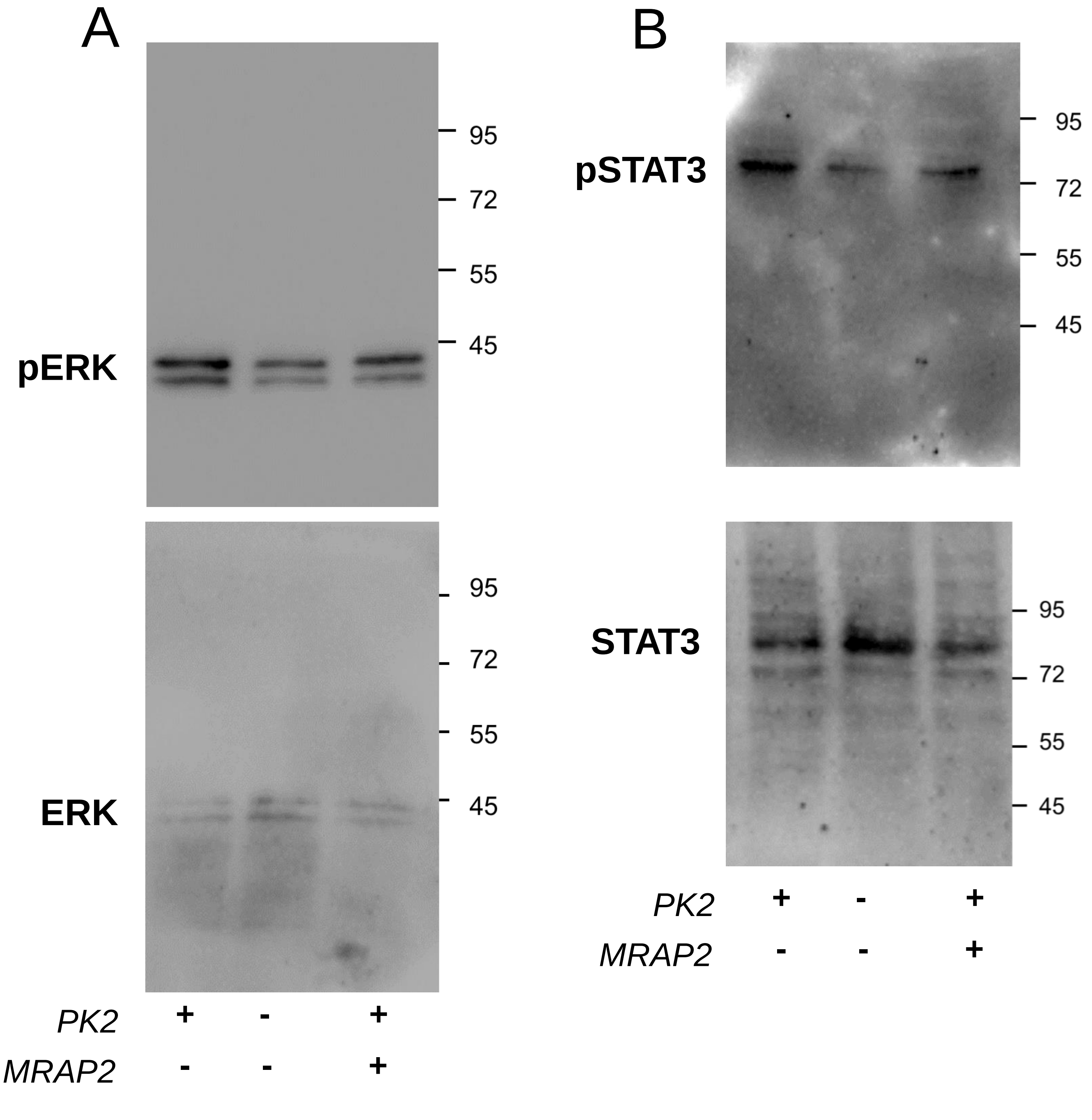

Figure S2. Original western blots of figure 5

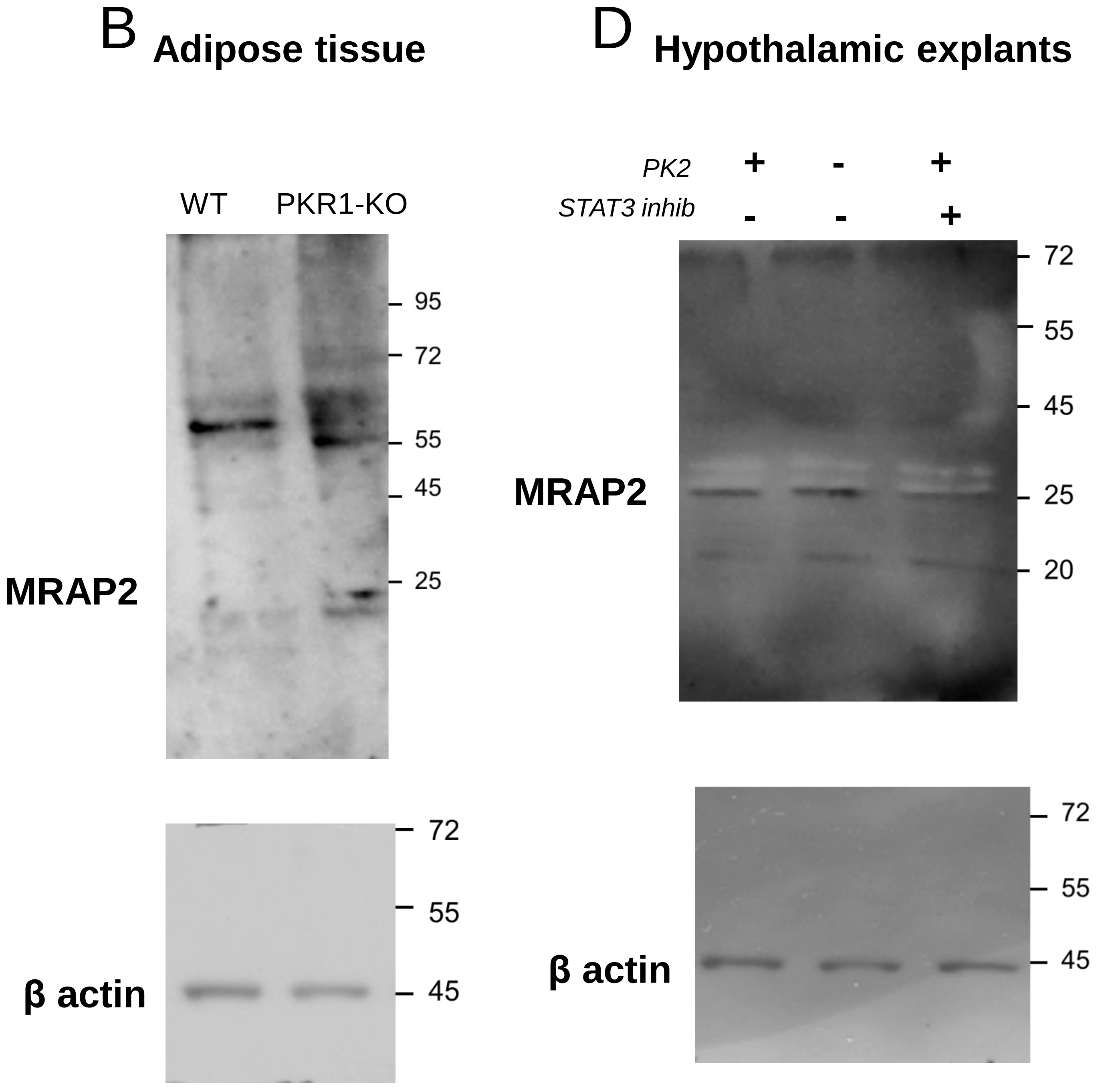

Figure S3. Expression of MRAP2 in different mouse tissues. 10 ug of total proteins were analyzed western blot analysis. S: skin; C: colon; A: adypocytes; B:brain

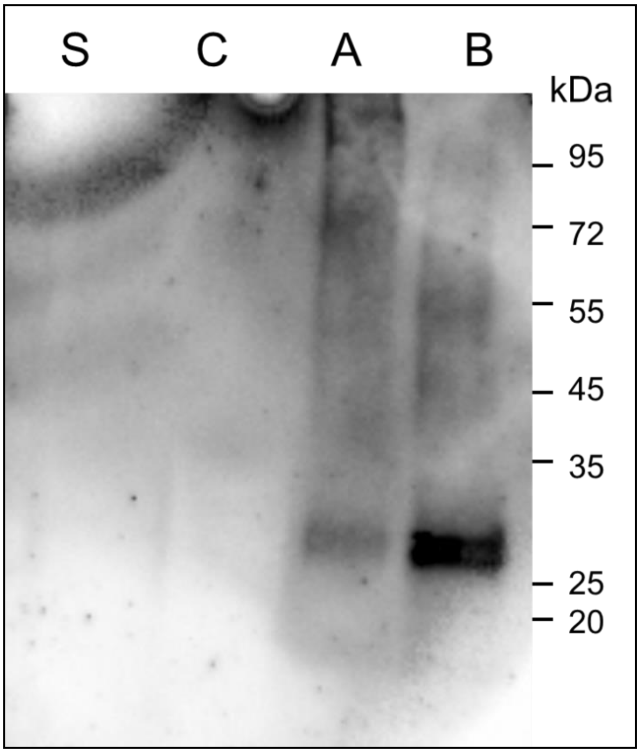

Figure S4. Cross-linking of MRAP2 with PKR2 or ΔN-PKR2 receptors. Membrane proteins prepared from *S. cerevisiae* cells expressing the PKR2-MRAP2 and ΔN-PKR2 -MRAP2 were incubated in the presence (+) or absence (−) of BS^3. Proteins were immunoblotted and probed with anti-PKR2 antibody. The arrow indicates the complex PKR2/MRAP2

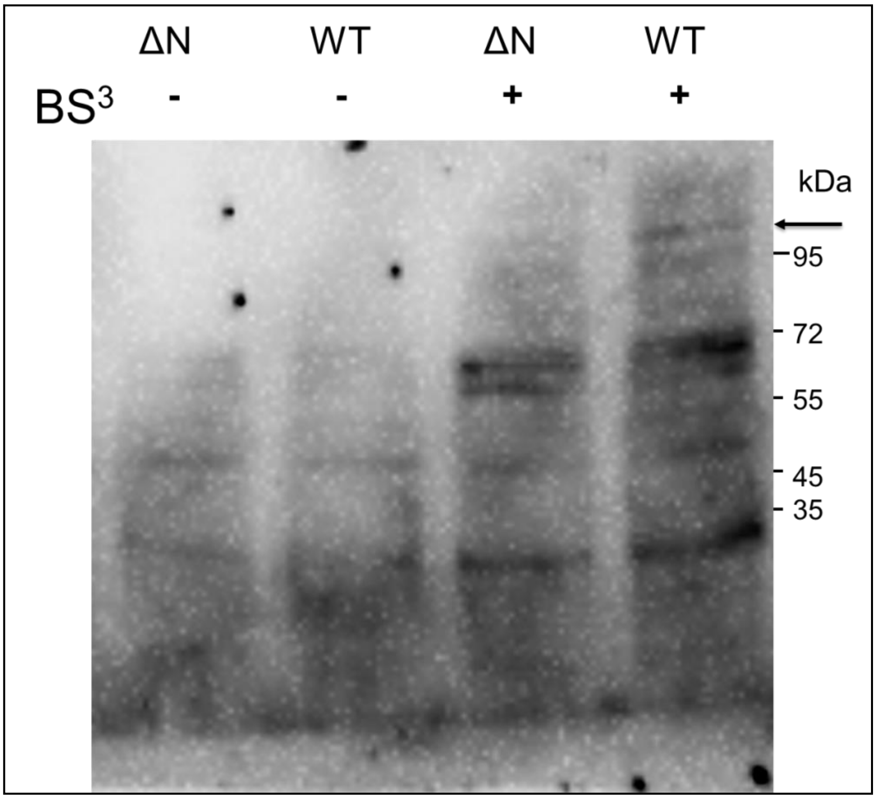

Supplement: Supplementary file 1 [file biomolecules-12-00474-s001.zip › biomolecules-1605974-supplementary.pdf]
